# Physical activity monitoring using wearable devices based on machine learning algorithms

**DOI:** 10.3389/fspor.2026.1817383

**Published:** 2026-04-24

**Authors:** Zhen Zhang, Yanxi Ren, Jin Zeng, Quan Ma

**Affiliations:** 1 School of Intelligent Sports Engineering, Wuhan Sports University, Wuhan, China; 2 School of Information Management, Wuhan University, Wuhan, China; 3 School of Nursing, Wuhan University, Wuhan, China; 4 School of Information Management, Hubei University of Economics, Wuhan, China

**Keywords:** energy expenditure estimation, metabolic equivalents (METs), physical activity monitoring, sleep stage detection, wearable accelerometry

## Abstract

Wearable accelerometers provide an important source for continuous physical activity monitoring, but high-frequency signals often suffer from missing labels, temporal discontinuity, and inter-individual variability, limiting their direct use in behavioral health analysis. This study proposes an integrated framework for wrist-worn tri-axial accelerometer data. The dataset includes 100 participants under free-living conditions, each monitored for approximately 24–27 h at 100 Hz. The framework consists of three stages: (1) data quality processing with label continuity reconstruction, (2) metabolic equivalent (MET) estimation using an XGBoost regression model based on time- and frequency-domain features, and (3) behavioral interpretation using the predicted MET sequence to derive sleep-stage boundary segmentation and sedentary-event alerts. XGBoost achieved the best performance among the evaluated models (MAPE = 0.2895, MSE = 0.6032). The method effectively captures temporal activity patterns, enabling sleep-stage transition identification and sedentary-event detection (MET < 1.6 for 30 min). The proposed framework provides an interpretable mapping from accelerometer signals to behavioral-health indicators, offering a practical approach for long-term wearable-based monitoring and personalized health management.

## 1 Introduction

Physical activity (PA) is commonly defined as any bodily movement produced by skeletal muscles that leads to energy expenditure [1]. PA can be assessed through multiple approaches, including direct observation, energy metabolism measurements (e.g., indirect calorimetry and doubly labeled water), and wearable sensing; however, these methods differ substantially in scalability, cost, and temporal resolution [2]. Gold-standard approaches are often limited by cost and implementation constraints, making them unsuitable for large-scale, long-term monitoring [3]. In contrast, accelerometer-based continuous recording provides a scalable and objective means to characterize daily activity patterns and sedentary behavior, supporting early detection of chronic disease risks [4]. Moreover, sedentary behavior and low activity levels have been associated with increased risks of adverse health outcomes such as cardiovascular disease and diabetes [5], while sleep—also measurable using wrist-worn devices—has been identified as an independent risk factor for cardiovascular disease and cancer [6].

Metabolic equivalent of task (MET) is widely used as a standardized index to quantify activity-related energy expenditure, enabling comparison across activities and supporting personalized health guidance [7]. Although indirect calorimetry remains an important reference for energy expenditure measurement, its practical limitations restrict its use in free-living conditions [8]. Accelerometers provide high-resolution motion signals with low burden and have become core sensors for estimating activity intensity in real-world settings [9]. Among different wearing locations, wrist-worn devices offer advantages in compliance and continuous 24-h monitoring, making them increasingly adopted in large-scale studies [4, 10]. However, wrist signals are more sensitive to upper-limb movements unrelated to whole-body motion, which may introduce estimation bias and increase the demand for robust analytical methods [11].

Machine learning approaches have been widely applied to high-frequency wearable time-series data to extract informative representations for activity recognition and energy estimation [12]. Compared with rule-based methods, they better capture complex patterns across behaviors and improve intensity estimation performance [13]. Tree-based models, in particular, offer a practical balance between interpretability and predictive ability for regression tasks [14]. In addition, preprocessing steps such as handling missing data, non-wear periods, and noise are critical for ensuring reliable downstream modeling [15]. Beyond intensity estimation, wrist-accelerometer data have also been used for sleep analysis and sedentary monitoring, where temporal structure—such as duration and continuity—plays a key role [16, 17].

Based on the above, this study develops an interpretable analytical pipeline that maps continuous wrist-worn accelerometer signals to behavioral-health indicators. The framework integrates data preprocessing, MET estimation, and behavioral interpretation within a unified workflow for long-term monitoring. Specifically, it includes: (1) data quality assessment and label-continuity reconstruction for high-frequency time series; (2) a feature-based machine learning regression model for MET estimation; and (3) a behavioral interpretation module for detecting sleep-stage transitions and sedentary events. The main contributions are threefold: (1) a unified pipeline linking raw signals to interpretable indicators; (2) a feature-driven MET regression model using multi-domain features; and (3) practical algorithms for sleep segmentation and sedentary alerting suitable for wearable systems.

## 2 Related work

### 2.1 MET and energy expenditure estimation from accelerometer signals

MET and energy expenditure estimation are core problems in physical activity quantification; they can be used for intensity grading and also support personalized exercise prescriptions and health interventions. Traditional methods often use empirical regression or piecewise linear models based on “count thresholds,” but they can show systematic bias under free-living data and mixed complex behaviors. In recent years, researchers have emphasized regression estimation using raw tri-axial acceleration signals and derived features to improve adaptability to diverse behaviors. Studies on wrist-worn data show that energy expenditure estimation varies significantly across individuals and activity types, suggesting the need for models with stronger representation capacity to approximate nonlinear mappings [18]. Meanwhile, many studies in free-living or semi-free-living settings indicate that wrist signals are strongly affected by upper-limb movements, and without appropriate modeling strategies, estimation errors may accumulate in the moderate-to-vigorous intensity range [19]. Taken together, this evidence has driven a paradigm shift from “threshold/linear regression” to “feature-based + machine learning regression” [20].

At the model level, tree models and their gradient-boosting variants are widely used for energy expenditure estimation because of their strong fit to structured features, ability to model nonlinear relationships, and good training stability. Recent work shows that for energy expenditure estimation using wrist-worn signals, personalized or stratified calibration strategies can further improve error performance under cross-population conditions [21]. In addition, studies using gradient-boosting frameworks often achieve better or more robust regression performance in multi-dataset benchmarks, especially when the feature dimension is large and the data distribution is imbalanced [5]. However, most published studies still point out that sparse samples in high-MET ranges and long-tailed distributions of activity types limit the model upper bound, making “data and feature representation” one of the performance bottlenecks [22]. Therefore, a key trend in current MET estimation research is to build robust feature sets and use strong nonlinear regressors while maintaining interpretability, and to explicitly consider the error structure caused by distribution imbalance in evaluation [23].

### 2.2 Sleep detection and stage-structure analysis from wrist-worn data

Wrist-worn accelerometers have long been used for sleep–wake estimation and have supported studies of sleep habits and health outcomes in large cohorts. With the accumulation of algorithms and data, researchers have expanded from macro indicators such as “sleep duration/efficiency” to the assessment of sleep timing, rhythms, and segmented structure. Studies based on the UK Biobank show that sleep onset time inferred from wrist-worn accelerometers is associated with cardiovascular disease incidence, suggesting the potential value of wearable sleep parameters for risk stratification [24]. Meanwhile, studies on sleep-stage recognition have continued to emerge, especially those using multi-sensor data or deep learning models to improve discrimination among light sleep, deep sleep, and REM stages [25]. Recent reviews further indicate that sleep-stage models show substantial heterogeneity in data collection protocols, labeling sources, and evaluation metrics, and generalization across devices and populations remains a major challenge [26]. Therefore, in resource-limited settings or applications that do not require complex training, it remains practically important to stably characterize sleep-structure boundaries using low-cost methods [27].

Beyond deep learning approaches, traditional signal processing and rule/threshold methods still play a role in sleep-structure boundary detection, especially for robust localization of stage transition points. For long time-series wrist-worn signals, stage transitions often appear as abrupt changes in activity level or structural changes in statistical features, so “change-point detection” has become an interpretable modeling perspective. Related studies show that using change features from acceleration differences or activity counts can enhance sensitivity to sleep–wake transitions and nocturnal disturbances, and can be used to construct visualized and interpretable segmentation results [5]. At the same time, considering the clear differences in signal scale and nocturnal activity background across individuals, adaptive or personalized threshold strategies help reduce false positives and false negatives caused by fixed thresholds [27]. Therefore, sleep analysis methods show a “two-track” pattern: on the one hand, data-driven models pursue end-to-end performance; on the other hand, lightweight strategies centered on differencing, thresholds, and segmentation emphasize interpretability and deployability [25].

### 2.3 Sedentary behavior detection, thresholds, and alert strategies

Sedentary behavior is usually defined as sitting/lying still with low energy expenditure and has been confirmed to be associated with various adverse cardiometabolic outcomes; therefore, identifying sedentary patterns from wearable data has clear public health value. Recent accelerometer-based studies further suggest that the relationship between sedentary time and cardiovascular outcome risk may follow a “threshold-type” dose relationship, providing a basis for interventions such as reducing prolonged sedentary bouts [25]. Meanwhile, in older women, device-measured sedentary and active time have been used to predict heart failure risk, highlighting the application potential of sedentary identification for health management in high-risk populations [28]. Methodologically, sedentary identification can be based on MET thresholds, activity count thresholds, or posture/context fusion inference; among them, MET thresholds (e.g., 1.5–1.6) are often used for behavior definition and interpretable outputs because they are directly related to energy expenditure intensity [29]. However, sedentary behavior has temporal characteristics that combine persistence and fragmentation, and point-wise threshold decisions are often insufficient to stably describe “continuous sedentary events” [30].

Therefore, researchers usually introduce temporal aggregation mechanisms to extend sedentary identification from “point-wise classification” to “event detection.” For implementing event detection, sliding windows and continuous-segment decisions are common and easy-to-deploy strategies: by aggregating low-intensity windows along the timeline, they identify sedentary events that meet duration criteria and output event start/end points to support alerts. Compared with only counting total daily sedentary time, event-level outputs can describe the timing distribution and frequency of sedentary behavior, providing more actionable trigger conditions for personalized interventions [19]. At the same time, to avoid repeated alerts for the same continuous sedentary period, the literature often uses mechanisms such as “post-trigger skip steps/cool-down time” to reduce redundant warnings and improve usability [30]. Notably, the effectiveness of sedentary alert models depends not only on threshold and duration definitions but also on the error structure of upstream MET estimation or intensity decisions; therefore, sedentary detection and energy-intensity modeling are coupled in the methodological pipeline [5]. In summary, an important trend in sedentary research is to combine energy-intensity estimation with event-level sedentary detection within a unified 24-h wrist-worn data framework, to support a closed loop from measurement to alerting.

## 3 Dataset

The dataset used in this study was provided by the 2025 (13th) “Teddy Cup” Data Mining Challenge. The recordings were collected from wrist-worn tri-axial accelerometers worn by volunteers under free-living conditions, rather than in a controlled laboratory environment. Participants wore the devices continuously during daily activities and nighttime sleep, enabling the capture of natural behavioral patterns.The dataset includes sex, age, and time information for 100 volunteers, as well as accelerometer data and the corresponding metabolic equivalent (MET) data. The dataset is a time series with a resolution of 0.01s. Each wristband tri-axial accelerometer record contains timestamped acceleration signals on the x, y, and z axes, as well as annotation fields describing activity status and the corresponding MET values, as shown in Table 1.

**Table 1:** Data file format.

Time	x	y	z	Annotation
Millisecond-level timestamp	Triaxial acceleration along X-axis (g)	Triaxial acceleration along Y-axis (g)	Triaxial acceleration along Z-axis (g)	Label containing MET value

The annotation field contains the activity type at the corresponding time point and its associated MET value (e.g., “sleeping; MET 0.95”). The MET value is extracted during preprocessing for subsequent analysis.

The accelerometer signals were sampled at 100 Hz, corresponding to a time resolution of 0.01 s between samples. For each participant, the recordings cover approximately 24–27 h of continuous monitoring, spanning a full day–night cycle. This recording duration includes sleep, sedentary periods, and various levels of physical activity, making the dataset suitable for evaluating wearable-based behavioral monitoring algorithms.

In addition to acceleration signals, the dataset also includes basic demographic attributes, namely age group and sex. According to the dataset specification, age is divided into four ranges (18–29, 30–37, 38–52, and ≥ 53 years), and sex is labeled as male (M) or female (F).

The annotation field provides activity descriptions and corresponding MET values as an objective indicator of physical activity intensity according to the principles of MET-based intensity classification shown in Table 2. However, the annotation sequence contains intermittently missing segments, which usually occur between two adjacent activity states. Therefore, systematic quality assessment and label-continuity analysis were conducted before model development.

**Table 2:** Annotation interpretation based on MET thresholds.

Activity intensity level	MET range	Description
Vigorous	MET ≥ 6.0	High-intensity physical activity
Moderate	3.0 ≤ MET < 6.0	Moderate-intensity physical activity
Light	1.6 ≤ MET < 3.0	Light-intensity activity
Sedentary	1.0 ≤ MET < 1.6	Sedentary behavior
Sleep	MET < 1.0	Sleep state

The MET-based intensity classification follows the internationally recognized standard from the Compendium of Physical Activities [31].

The dataset from 100 volunteers was mainly used for model development and feature learning. In addition, a dataset containing 20 volunteers was reserved for independent prediction and validation of the MET estimation model.

## 4 Methods

**Figure 1:**
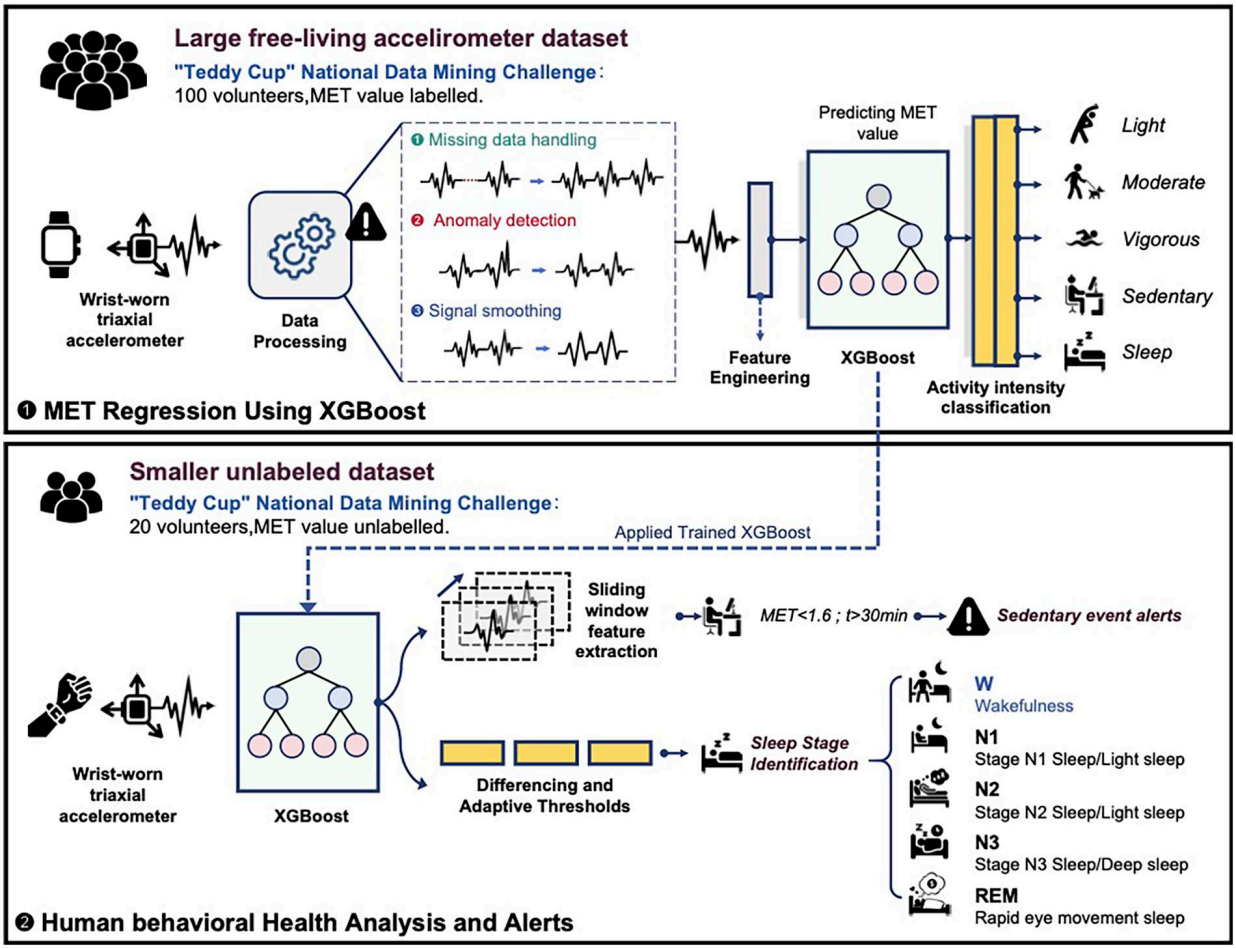
A frame diagram of a wearable device for physical activity monitoring based on machine learning algorithms.

The overall workflow of a wearable device for physical activity monitoring using a machine learning algorithm is shown in Figure 1.

### 4.1 Key parameters used in the analytical pipeline

To improve clarity and reproducibility, the key parameters used in the analytical pipeline are summarized in Table 3. These parameters include window segmentation settings, sedentary detection thresholds, and coefficients used in the sleep-stage segmentation algorithm.

**Table 3:** Model parameters used in the study.

Parameter	Symbol	Value	Description
Sampling frequency	f	100 Hz	Accelerometer sampling rate
Window length	L	10,000 samples	Feature extraction window
Step size	S	10,000 samples	Sliding window step
Sedentary duration threshold	$W_{s}$	18 windows	Equivalent to 30 min
Sleep threshold scale	k	empirical	Threshold sensitivity parameter
Dynamic threshold coefficient	λ	empirical	Adaptive threshold update coefficient

### 4.2 Data quality assessment and annotation-structure analysis

To ensure the reliability of subsequent modeling results, we first performed batch quality checks on the temporal consistency, duplicate records, and missingness of the raw sensor data. On this basis, for missing segments in the annotation column, we developed an unsupervised anomaly detection and imputation strategy based on duration statistics and DBSCAN density clustering to obtain a continuous representation along the timeline. First, we conducted batch detection of duplicate records and missingness, and the related processing used the Polars framework for data loading and quality checking. The results showed that the time interval between adjacent records was 0.01 s, corresponding to a sampling rate of 100 Hz, and no duplicate records were found. During the data quality assessment stage, we observed intermittent missing annotation segments in a small number of participants (P004, P049, P057, P073, and P077). These missing segments typically occurred between two adjacent activity states, which is consistent with short acquisition interruptions or temporary annotation gaps during device recording. Overall, the proportion of missing records was less than 1% of the total dataset, indicating that the dataset maintains high temporal completeness. Because these missing segments are short and sparsely distributed, we treated them as brief acquisition-stage interruptions rather than systematic measurement errors. To ensure temporal continuity for modeling, we reconstructed the annotation sequence using a duration-statistics-based clustering strategy combined with DBSCAN density clustering, which allows adjacent activity segments to be connected while preserving the overall temporal structure.

First, we calculated the durations of consecutive missing segments in the annotation column and constructed a duration set, denoted as $T=[t_{1},t_{2},\ldots ,t_{k}]$. We then performed clustering analysis on this set using DBSCAN. The decision rules of DBSCAN are as follows:

Core point: For a data point p, if its neighborhood | contains at least MinPts points, then p is a core point:

CorePoint(p)↔|{q∣dist(p,q)≤ε}|≥MinPts. (1)

Reachable point: Point q is reachable from point p if and only if there exists a sequence of core points $p_{1},p_{2},\ldots ,p_{k}$ such that every core point on the path from $p_{i}$ to $p_{k}$ lies within the |-neighborhood.

Noise point: If a point is neither a core point nor reachable from any core point, it is labeled as a noise point. Anomaly detection: By clustering the duration list T with DBSCAN, anomalous intervals that do not belong to any cluster can be identified. The results showed that most missing durations were less than 20 min and were mainly distributed near activity-state transitions. Further inspection found that missing values in the annotation column were distributed in phases along the timeline. Accordingly, we treated the missing intervals as transition buffers between adjacent activity states. Under this assumption, we imputed missing segments using the mean MET of the nearest non-missing intervals before and after the gap.

To enable numerical processing, we extracted MET values from the annotation field using the regular expression ;MET (∖d+∖.∖d+), (where ; is a literal semicolon). We then performed imputation using the mean of forward-fill and backward-fill values, as shown below:

$$x_{i}=\left\{ \begin{array}{cc} \frac{\text{ForwardFill}(x_{i})+\text{BackwardFill}(x_{i})}{2},\; & x_{i}=\text{null},\;\\ x_{i},\; & x_{i}\neq \text{null}.\; \end{array}\right.$$ (2)

For consecutive missing intervals at the end, we corrected them using forward fill only.

### 4.3 MET statistics

Based on the above quality assessment, the sampling rate was stable at 100 Hz. Therefore, the time duration can be directly converted from the sample count by dividing the count by 360,000 to obtain hours. Based on this, the MET statistics workflow is as follows:

MET value extraction: First, we parsed the annotation field in a structured manner and extracted the corresponding MET values using the regular expression ;MET (∖d+∖.∖d+), (where ; is a literal semicolon) to construct a continuous MET series:

$${\text{MET}}_{i}=f({\text{annotation}}_{i}),$$ (3)

where ${\text{MET}}_{i}$ denotes the metabolic equivalent at time point i.

**Missing-interval imputation:** For missing intervals in the annotation field, we used a segment-wise imputation strategy based on the mean of adjacent states. Let the valid MET values adjacent to a missing interval be ${\text{MET}}_{\text{\textbackslash{},prev}}$ and ${\text{MET}}_{\text{next}}$; the imputed value for this interval is:

$${\text{MET}}_{\text{\textbackslash{},fill}}=\frac{{\text{MET}}_{\text{\textbackslash{},prev}}+{\text{MET}}_{\text{next}}}{2},$$ (4)

For consecutive missing segments at the end of the recording, we used forward fill only:

$${\text{MET}}_{\text{\textbackslash{},fill}}={\text{MET}}_{\text{\textbackslash{},prev}},$$ (5)

After imputation, the annotation yields a continuous, computable MET time series along the timeline.

Grouped by MET level: After constructing a continuous MET series, we grouped samples by MET level and counted valid records for each intensity level:

$$N_{k}=\sum_{i=1}^{T}1({\text{MET}}_{i}=k),$$ (6)

$N_{k}$ is the sample count for MET level k, T is the total number of samples, and 1(⋅) is an indicator function.

Time conversion: At 100 Hz, the theoretical number of samples per hour is:

$$F_{\mathrm{hour}}=100\times 60\times 60=360,000,$$ (7)

Therefore, the duration (hours) for each MET level is computed as:

$$t_{k}=\frac{N_{k}}{360,000},$$ (8)

$t_{k}$ denotes the cumulative duration (hours) at MET level k.

We then used a Polars-based multithreaded pipeline to batch summarize the 100 accelerometer data. Because converting sample counts to time may introduce rounding errors, we computed the mean time deviation:

$$\Delta t=\left|t_{\mathrm{count}}-t_{\mathrm{timestamp}}\right|,$$ (9)

This yielded:

Δt=0.00011, (10)

This workflow generates a continuous MET series and summarizes the distribution of activity intensity.The mean time difference was 0.00011, which is within an acceptable tolerance.To illustrate the pipeline output, we present one participant (P100) as a representative example and apply the same procedure to the full cohort of 100 participants, as shown in Table 4.The final activity-time distribution is reported in Table 5.

**Table 4:** Example output of activity duration distribution for participant P100 after the unified processing pipeline (hours).

Participant ID	Total recording time	Sleep	Sedentary	Light	Moderate	Vigorous
P100	26.4167	7.25	7.6898	5.1997	6.1807	0.0964

**Table 5:** Activity time statistics.

Age	Mean total recording time	Mean total sleep time	Mean vigorous activity	Mean moderate activity	Mean light activity	Mean sedentary activity
18–29	26.5288	7.3947	0.1008	1.7941	6.0065	11.2328
30–37	26.6094	7.5308	0.0227	2.1973	6.6770	10.1816
38–52	26.0770	6.4330	0.0483	1.8934	6.6946	11.0077
53+	26.5417	6.1013	0.0423	2.8983	5.6606	11.8392

### 4.4 MET prediction

#### 4.4.1 Data preprocessing

To improve the stability and reliability of MET prediction from wrist-worn accelerometer data, we preprocessed the raw acceleration signals before model development.The preprocessing pipeline included missing-value handling, outlier detection, and signal smoothing.

First, we processed missing values in the raw data.Because MET prediction requires continuous time-series signals, we removed records with missing values and retained only complete accelerometer data for subsequent analysis and modeling.

Next, we applied the Isolation Forest algorithm to detect anomalies in the tri-axial acceleration data, in order to identify and handle abnormal samples that may be caused by sensor noise or atypical wearing conditions.By computing the average path length of each sample across multiple isolation trees, we obtained an anomaly score; samples with higher scores were identified as outliers and handled as follows:

Here, S(x) denotes the anomaly score of sample x, E(h(x)) denotes its mean path length across isolation trees, and c(n) is a normalization constant that depends on the dataset size n.

After anomaly detection, we replaced identified outliers with neighborhood-mean values to preserve time-series continuity.We then smoothed the acceleration signals using a moving-average filter to further suppress high-frequency noise.We used a window size of 10, computing the mean over the current point and five neighboring points on each side.

**Figure 2:**
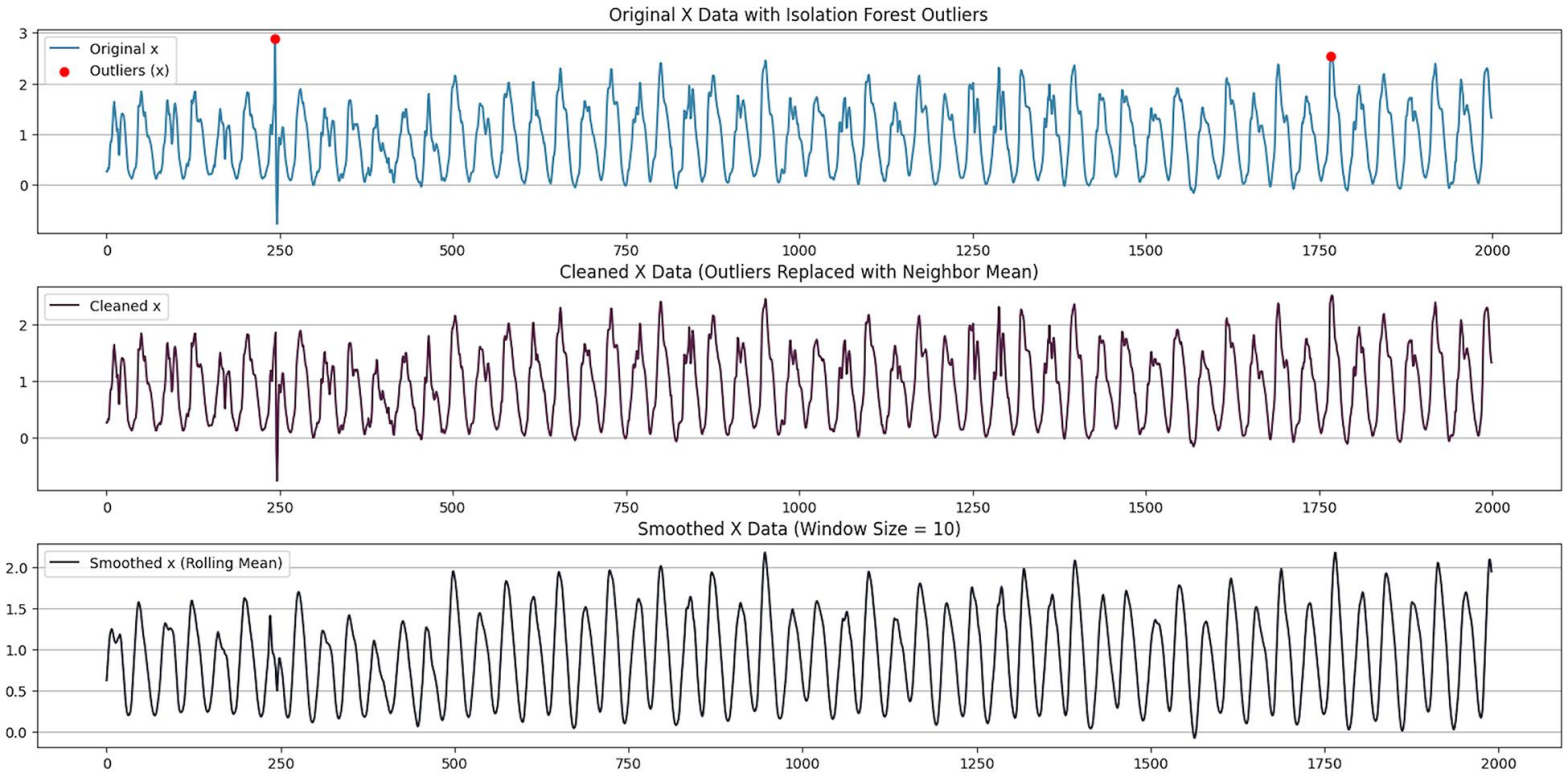
Data anomaly handling and filtering.

The smoothing operation is defined as follows:

$$\left\{ \begin{array}{c} \{x_{1}^{\prime },x_{2}^{\prime },\ldots ,x_{n}^{\prime }\}=\text{Isolation}\;\left(\{x_{1},x_{2},\ldots ,x_{n}\}\right)\\ y_{n}=\frac{1}{w}\sum_{t=n-\frac{w}{2}}^{\;n+\frac{w}{2}}x_{t}\\ y_{n}=\frac{1}{10}\left(x_{n-5}^{\prime }+x_{n-4}^{\prime }+\cdots +x_{n+4}^{\prime }\right) \end{array}\right.,$$ (11)

where w denotes the window size and $x_{i}$ denotes the data point. Using x acceleration axis as an example, Figure 2 compares the raw signal, the signal after outlier handling, and the signal after moving-average smoothing.After outlier handling and smoothing, spikes and noise were substantially reduced while key movement patterns were preserved, providing higher-quality inputs for MET prediction.

#### 4.4.2 Feature engineering

To fully explore information in wrist-worn acceleration signals related to physical activity intensity and convert continuous time-series data into structured representations suitable for machine learning models, we constructed a set of multidimensional features based on the preprocessed tri-axial acceleration signals. The extracted features mainly include time-domain statistical features, frequency-domain features, inter-axis correlation features, and basic individual information features.

Time-domain statistical features: Time-domain statistics describe the overall distribution and variability of acceleration signals within local time windows. We extracted the mean and standard deviation from acceleration signals in each time window as basic statistical features. The mean reflects the overall level of the signal:

$$\mu_{x}=\frac{1}{N}\sum_{i=1}^{N}x_{i},$$ (12)

$x_{i}$ denotes the signal value at time index i.

The standard deviation quantifies signal variability:

$$\sigma_{x}=\sqrt{\frac{1}{N}\sum_{i=1}^{N}{\left(x_{i}-\mu_{x}\right)}^{2}},$$ (13)

$\mu_{x}$ is the signal mean and $x_{i}$ is the value at time index i.

Frequency-domain features: After transforming the signal to the frequency domain using the fast Fourier transform (FFT), we extracted features that capture activity-related rhythmic patterns.We applied FFT to the acceleration signals and extracted representative frequency-domain features.Specifically, we computed the dominant frequency, spectral amplitude, and spectral centroid.

The dominant frequency is the component with the maximum amplitude:

$$f_{\max}=\arg\max\left|\mathrm{FFT}(x)[i]\right|,$$ (14)

x is the signal, FFT(x) is its Fourier transform, and $f_{\max}$ is the frequency with the maximum amplitude.

Spectral amplitude reflects the energy of the dominant frequency components:

$$\mathrm{SpecAmp}=\sum_{i=0}^{N/2}\left|\mathrm{FFT}(x)[i]\right|,$$ (15)

N is the signal length and FFT(x)[i] is the i-th FFT coefficient.

The spectral centroid summarizes where spectral energy is concentrated:

$$f_{\mathrm{center}}=\frac{\sum_{i=0}^{N/2}f_{i}\;\left|\mathrm{FFT}(x)[i]\right|}{\sum_{i=0}^{N/2}\left|\mathrm{FFT}(x)[i]\right|},$$ (16)

$f_{i}$ is the i-th frequency and |FFT(x)[i]| is its amplitude.

Inter-axis correlation features: Different activities exhibit distinct coordination patterns across the three acceleration axes.To quantify coupling across axes, we computed Pearson correlation coefficients for each pair among the x, y, and z axes.Correlations were computed by normalizing covariance by the standard deviations of the two axes, as defined below:

$$\left\{ \begin{array}{cc} \mathrm{corr}(x,y) & =\frac{\mathrm{cov}(x,y)}{\sigma_{x}\sigma_{y}}\\ \mathrm{corr}(x,z) & =\frac{\mathrm{cov}(x,z)}{\sigma_{x}\sigma_{z}}\\ \mathrm{corr}(y,z) & =\frac{\mathrm{cov}(y,z)}{\sigma_{y}\sigma_{z}} \end{array}\right.,$$ (17)

where cov(x,y) is the covariance between x and y, and $\sigma_{x}$ and $\sigma_{y}$ are their standard deviations (similarly for other axis pairs).

Basic participant features: At the population level, MET is closely related to an individual’s basal metabolic rate, and basal metabolic rate is influenced by physiological factors such as age and sex. Therefore, in addition to signal statistics and frequency-domain features, we included age and sex as participant-level auxiliary features to better capture metabolic differences across individuals and improve personalized MET prediction accuracy. According to the age-bin distribution, age was divided into four groups (18–29, 30–37, 38–52, and ≥ 53 years) and numerically encoded using a representative value for each group. Sex was encoded as a binary feature, with males coded as 1 and females coded as 0. The specific conversion rules are shown in Tables 6 and 7.

**Table 6:** Age data conversion.

Age range	Converted value
18–29	24
30–37	34
38–52	45
53+	53

**Table 7:** Gender data conversion.

Gender	Converted value
M	1
F	0

In summary, the final feature set is shown in Table 8, covering multi-dimensional features such as time-domain, frequency-domain, correlation, and demographic information.

**Table 8:** Feature set.

Feature	Formula
Mean	$\mu_{x}=\frac{1}{N}\sum_{i=1}^{N}x_{i}$
Variance	$\sigma_{x}=\sqrt{\frac{1}{N}\sum_{i=1}^{N}{\left(x_{i}-\mu_{x}\right)}^{2}}$
Maximum frequency	$f_{\max}=\arg\max\;\left|\mathrm{FFT}(x)[i]\right|$
Spectrum amplitude	$\mathrm{SpecAmp}=\sum_{i=0}^{N/2}\left|\mathrm{FFT}(x)[i]\right|$
Frequency centroid	$f_{\mathrm{center}}=\frac{\sum_{i=0}^{N/2}f_{i}\;\left|\mathrm{FFT}(x)[i]\right|}{\sum_{i=0}^{N/2}\left|\mathrm{FFT}(x)[i]\right|}$
Pearson correlation coefficient	$\{\begin{array}{c} \mathrm{corr}(x,y)=\frac{\mathrm{cov}(x,y)}{\sigma_{x}\sigma_{y}}\\ \mathrm{corr}(x,z)=\frac{\mathrm{cov}(x,z)}{\sigma_{x}\sigma_{z}}\\ \mathrm{corr}(y,z)=\frac{\mathrm{cov}(y,z)}{\sigma_{y}\sigma_{z}} \end{array}$
Age	–
Gender	–

#### 4.4.3 MET regression using XGBoost

To perform MET regression prediction based on accelerometer features, we selected XGBoost (Extreme Gradient Boosting) as the prediction model. XGBoost is an ensemble learning method based on gradient-boosted decision trees, which provides strong predictive performance and generalization for structured feature data.

Using the time-domain, frequency-domain, and correlation features constructed above, we formed samples $x=(x_{1},x_{2},\ldots ,x_{d})$, where d is the feature dimension, and XGBoost computes the final prediction via an ensemble of multiple trees. The prediction of each tree t is an additive model, expressed as:

$$f_{t}(x)=\sum_{i=1}^{T}{\hat{y}}_{i}\cdot I(x\in R_{i}),$$ (18)

T is the number of trees, ${\hat{y}}_{i}$ is the output of the i-th tree, and $I(x\in R_{i})$ is an indicator function that denotes whether sample x falls into leaf node $R_{i}$ of tree t.

Overall, the prediction process of XGBoost is an additive combination of multiple decision trees. Assuming there are M trees, the final prediction $\hat{y}$ is a weighted sum of the predictions from each tree:

$$\hat{y}(x)=\sum_{t=1}^{M}f_{t}(x)=\sum_{t=1}^{M}\sum_{i=1}^{T}{\hat{y}}_{i}\cdot I(x\in R_{i}).$$ (19)

Gradient boosting optimization: XGBoost optimizes the model using gradient boosting. Each new tree is fitted to the residuals of the current model. Suppose the current model prediction is ${\hat{y}}^{\left(k\right)}(x)$; at step k, XGBoost updates predictions by computing the residual $r_{i}^{\left(k\right)}$:

$$r_{i}^{\left(k\right)}=y_{i}-{\hat{y}}^{\left(k-1\right)}(x_{i}),$$ (20)

$y_{i}$ is the true MET value of the i-th sample.

Loss function and regularization: The XGBoost objective considers not only model error (e.g., mean squared error and log loss) but also a regularization term to prevent overfitting. The XGBoost objective can be written as:

$$L(\theta )=\sum_{i=1}^{N}\gamma \;\left(y_{i},{\hat{y}}_{i}\right)+\sum_{t=1}^{M}\Omega (f_{t}),$$ (21)

$\gamma \;(y_{i},{\hat{y}}_{i})$ is the loss function and $\Omega (f_{t})$ is the regularization term for the trees, which typically includes tree complexity (e.g., the number of leaf nodes and split points), and can be written as:

$$\Omega (f_{t})=\gamma T+\frac{1}{2}\lambda \sum_{k=1}^{T}\omega_{k}^{2},$$ (22)

T is the number of leaf nodes, γ and λ are regularization parameters, and $\omega_{k}$ is the weight of a leaf node.

**Figure 3:**
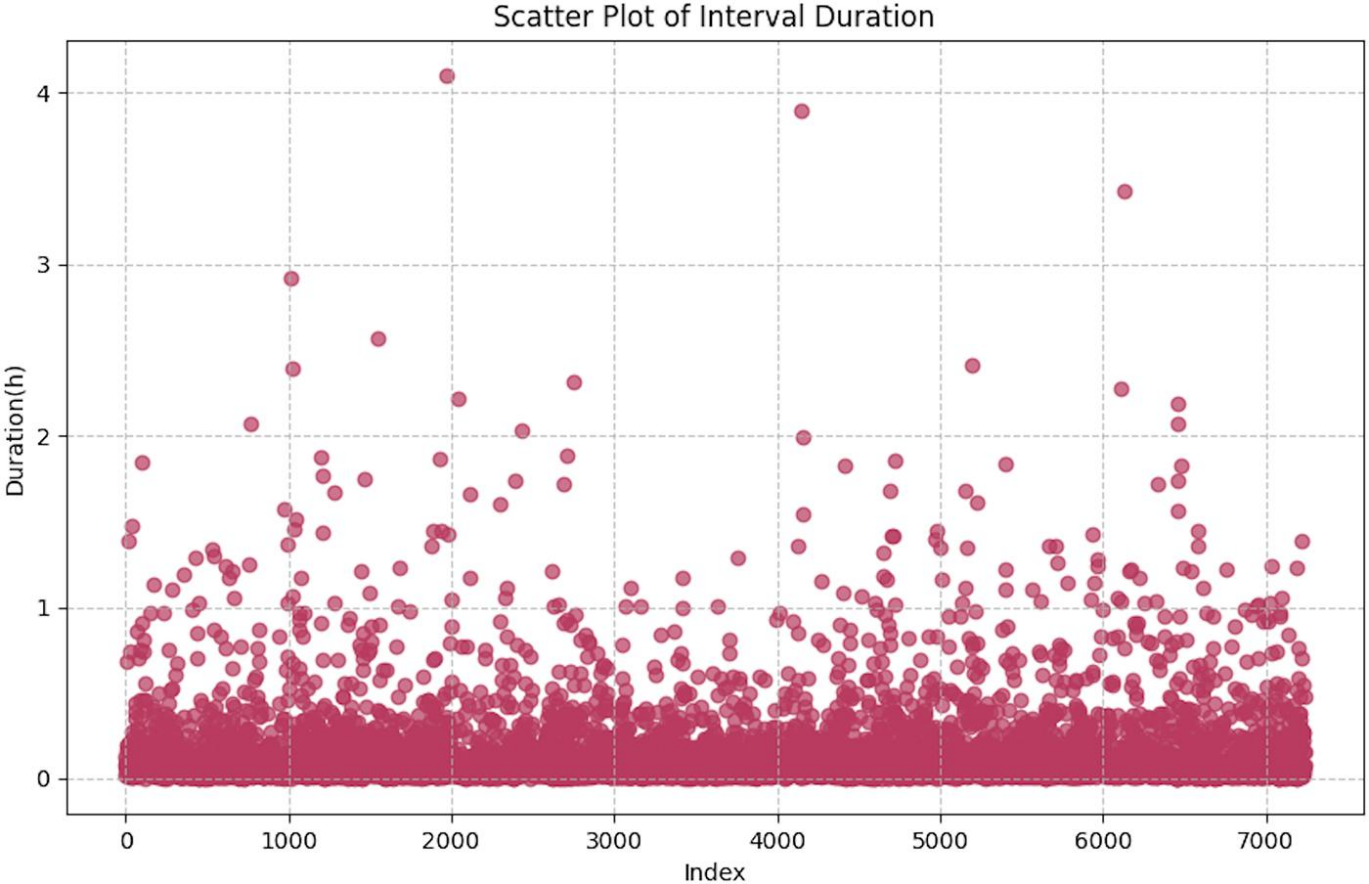
Scatter plot of the data window.

Window segmentation: Using wrist-worn accelerometer data from 100 volunteers in the training set, we analyzed and visualized the distribution of window lengths to guide the selection of sliding-window parameters, as shown in Figure 3. The statistics showed that the mean window duration was 0.1453 h, corresponding to approximately 52,308 samples.

Considering both predictive accuracy and computational efficiency, we selected 10,000 data points as the window step. For each window, we extracted statistical and frequency-domain features, used the resulting feature vector as model input, and the trained XGBoost model output the predicted MET value for the corresponding window.

### 4.5 Behavioral health analysis and alerts

#### 4.5.1 Sleep stage identification using differencing and adaptive thresholds

In this study, we used wrist-worn accelerometer data to automatically identify and analyze state changes during sleep. Combined with our earlier exploration of temporal-structure features in acceleration signals, we observed that sleep periods often show clear stage-like changes in the time series, with abrupt shifts in overall acceleration levels at stage transitions. Therefore, we formulated sleep-stage identification as a change-point detection task, characterizing sleep-state transitions by locating significant changes in the signal.

To achieve stable change-point localization, the method uses differencing to amplify signal changes and an adaptive-threshold strategy to dynamically adjust for scale differences across individuals, thereby enabling automatic sleep-stage boundary segmentation without complex model training. Using tri-axial acceleration signals as input, the method quantifies change intensity and applies thresholding to output stage segmentation results during sleep, providing a basis for subsequent sleep-structure analysis.

**Figure 4:**
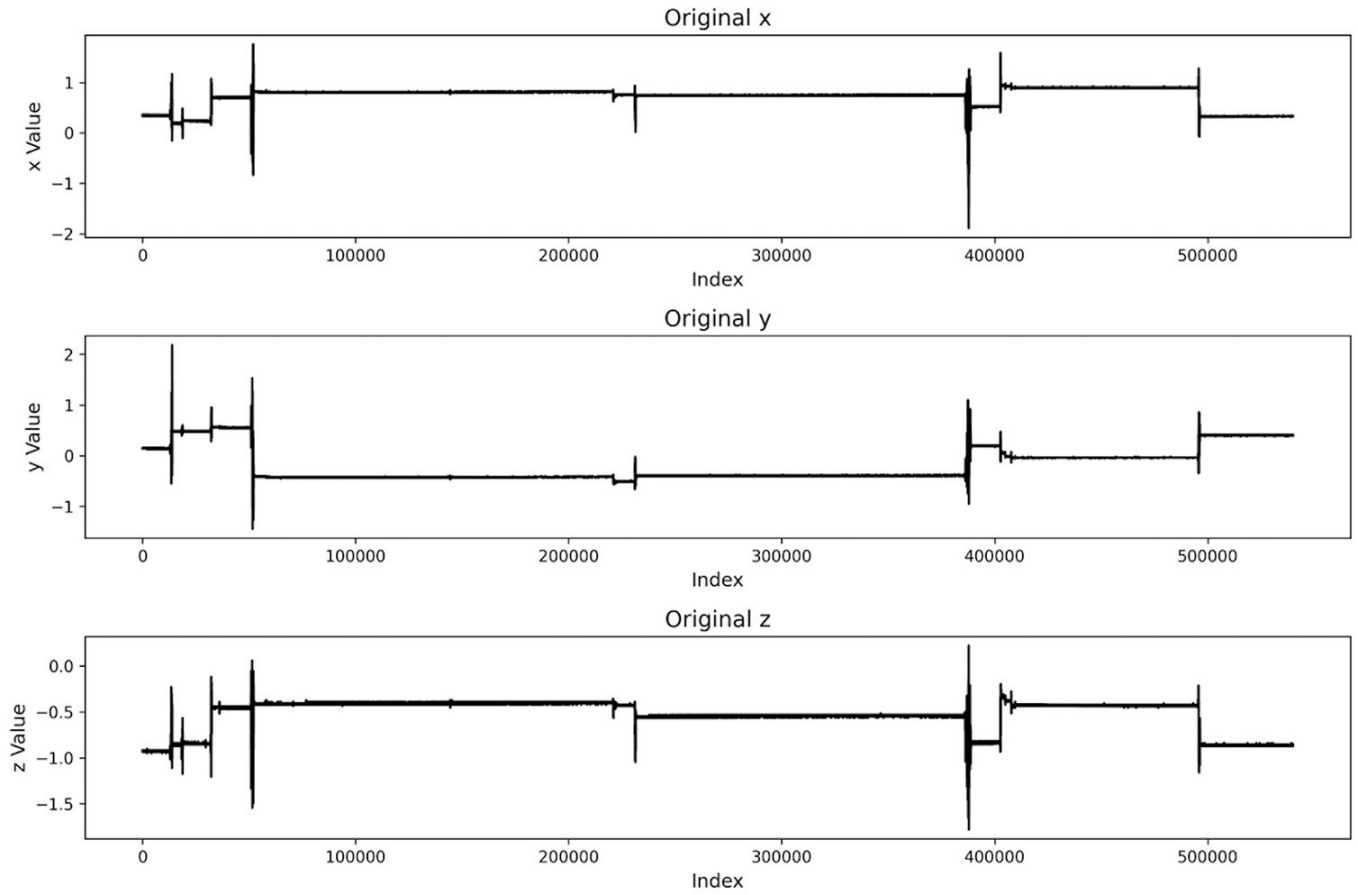
Differential visualization of data.

We conducted experiments using accelerometer data from 100 volunteers in the dataset. Using participant P096 as an example, we first visualized the MET changes during the sleep period, as shown in Figure 4.

It can be observed that after differencing, signal changes become more prominent and the positions of significant fluctuations are clearer. Based on this, a threshold is required to identify time points that may correspond to sleep-stage transitions. To adapt to inter-individual differences in signal amplitude, we used an adaptive-threshold strategy to dynamically adjust the decision criterion, as follows:

Differencing:

Δx(t)=x(t)−x(t−1), (23)

x(t) is the raw acceleration value at time t (one axis is used as an example), Δx(t) is the first-order difference between two adjacent time points, which quantifies instantaneous change, and t is the time index.

Statistical feature calculation:

$$\left\{ \begin{array}{cc} \mu_{\Delta x} & =\frac{1}{N}\sum_{t=1}^{N}\Delta x(t)\\ \sigma_{\Delta x} & =\sqrt{\frac{1}{N}\sum_{t=1}^{N}{\left(\Delta x(t)-\mu_{\Delta x}\right)}^{2}} \end{array}\right.,$$ (24)

where N is the number of points in the current analysis window, $\mu_{\Delta x}$ is the mean of the differenced series Δx(t), and $\sigma_{\Delta x}$ is its standard deviation, which measures the dispersion of change intensity.

Initial threshold:

$$\theta (t)=\mu_{\Delta x}+k\cdot \sigma_{\Delta x},$$ (25)

k is a scaling factor controlling threshold sensitivity; θ(t) is the adaptive threshold at timet, and $\theta_{t-1}$ is the threshold at the previous time point.

Dynamic threshold update:

$$\theta (t)=\theta_{t-1}+\lambda \cdot \max\left|\Delta x(i)\right|,i\in [1,t],$$ (26)

λ is an update coefficient for the dynamic threshold, and max|Δx(i)| is the maximum differenced magnitude observed over the interval [1,t].

Change-point detection:

Point tif|Δx(t)|>θ(t), (27)

i is a time index in [1,t]; when |Δx(t)|>θ(t), time t is identified as a potential sleep-stage change point.

The above method adaptively adjusts the threshold based on differenced changes and statistical features, thereby identifying significant change locations in the signal and determining sleep-stage boundaries. This process can maintain stable change-point detection performance across different individuals with different data scales.

#### 4.5.2 A sliding-window-based sedentary behavior alert model

Sedentary behavior is defined as remaining continuously in a static sitting posture with low energy expenditure. According to energy-expenditure intensity criteria, we defined a sedentary bout as MET < 1.6 sustained for more than 30 min. Prolonged sedentary behavior is strongly associated with increased risk of multiple chronic diseases; therefore, automatic detection and alerting based on wearable data are needed.

Using wrist-worn accelerometer data and corresponding activity annotations from 100 volunteers in the training set, we built a sedentary behavior detection model and applied it to data from 20 volunteers to output individual-level sedentary alerts.

Using the window length of 10,000 samples from the MET prediction stage, at a sampling rate of 100 Hz, 30 min corresponds to:

$$N_{30\;\text{min}}=30\times 60\times 100=180,000,$$ (28)

Therefore, one sedentary bout corresponds to:

$$W_{s}=\frac{180,000}{10,000}=18,$$ (29)

That is, a low-intensity state across 18 consecutive windows.

Sliding-window construction: Let the per-sample MET prediction sequence produced by XGBoost be $\left\{{\hat{y}}_{1},{\hat{y}}_{2},\ldots ,{\hat{y}}_{T}\right\}$, which is partitioned into windows of length L:

$$W_{k}=\{{\hat{y}}_{(k-1)L+1},\ldots ,{\hat{y}}_{(k-1)L+L}\},\;k=1,2,\ldots ,K,$$ (30)

L=10,000 is the window length and K is the total number of windows.

We computed the mean MET for each window:

$${\hat{y}}_{k}=\frac{1}{L}\sum_{i\in W_{k}}{\hat{y}}_{i}$$ (31)

where ${\hat{y}}_{k}$ denotes the mean MET value of the k-th window. Low-intensity labeling: Based on the sedentary threshold (1.6 MET), we defined a window-level state variable:

$$s_{k}=\left\{ \begin{array}{cc} 1,\; & {\hat{y}}_{k}<1.6\;\\ 0,\; & {\hat{y}}_{k}\geq 1.6\; \end{array}\right.,$$ (32)

$s_{k}=1$ indicates a low-energy state that may be sedentary, and $s_{k}=0$ indicates a non-sedentary state.

Continuous bout decision: We slid a window of length $W_{s}$ along the sequence $\left\{s_{k}\right\}$. If an interval satisfies:

$$\sum_{i=t}^{t+W_{s}-1}s_{i}=W_{s},$$ (33)

Then the interval $\left[t,t+W_{s}-1\right]$ is identified as a sedentary bout,and use the start time of window t as the bout onset.

Sedentary alerting: We defined the alert function as:

$$\text{Alert}(t)=\left\{ \begin{array}{cc} 1,\; & \sum_{i=t}^{t+W_{s}-1}s_{i}=W_{s}\;\\ 0,\; & \text{otherwise}\; \end{array}\right.,$$ (34)

Alert(t)=1 indicates a sedentary alert is triggered at time t, and Alert(t)=0 indicates that no sustained low-intensity behavior is detected.

To avoid repeated alerts during the same continuous sedentary period, we introduced a de-duplication rule. After an alert is triggered at time t, the algorithm enters a cool-down interval equal to the sedentary window length (Ws = 18 windows). During this interval, additional alerts are suppressed until the MET level rises above the sedentary threshold and a new sedentary bout begins. This mechanism ensures that each continuous sedentary episode generates only one alert event, improving interpretability and usability in real-world monitoring scenarios.

## 5 Experiments and evaluation

### 5.1 Data-processing efficiency comparison

To compare runtime efficiency across data-processing frameworks on large-scale accelerometer data, we conducted the following experiments.The experiments are described in Table 9:

**Table 9:** Experimental hardware configuration.

Component	Configuration
CPU	AMD Ryzen 5 5600 6-Core Processor
GPU	AMD Radeon RX 6500 XT
Memory	Crucial DDR4 3200 (16GB×2)
SSD	TiPlus 7100 Gen4 512GB

This configuration is at a relatively low hardware level.

#### 5.1.1 Full-dataset retrieval performance test

We used Pandas, Dask, and Polars to query accelerometer records from all participant files in dataset. The query condition was annotation = “7,030 sleeping; MET 0.95”.We measured the average query time for each framework under the same workload, using runtime as the efficiency metric. With an identical query, speed V is defined as the reciprocal of runtime T:

$$V=\frac{1}{T},$$ (35)

**Figure 5:**
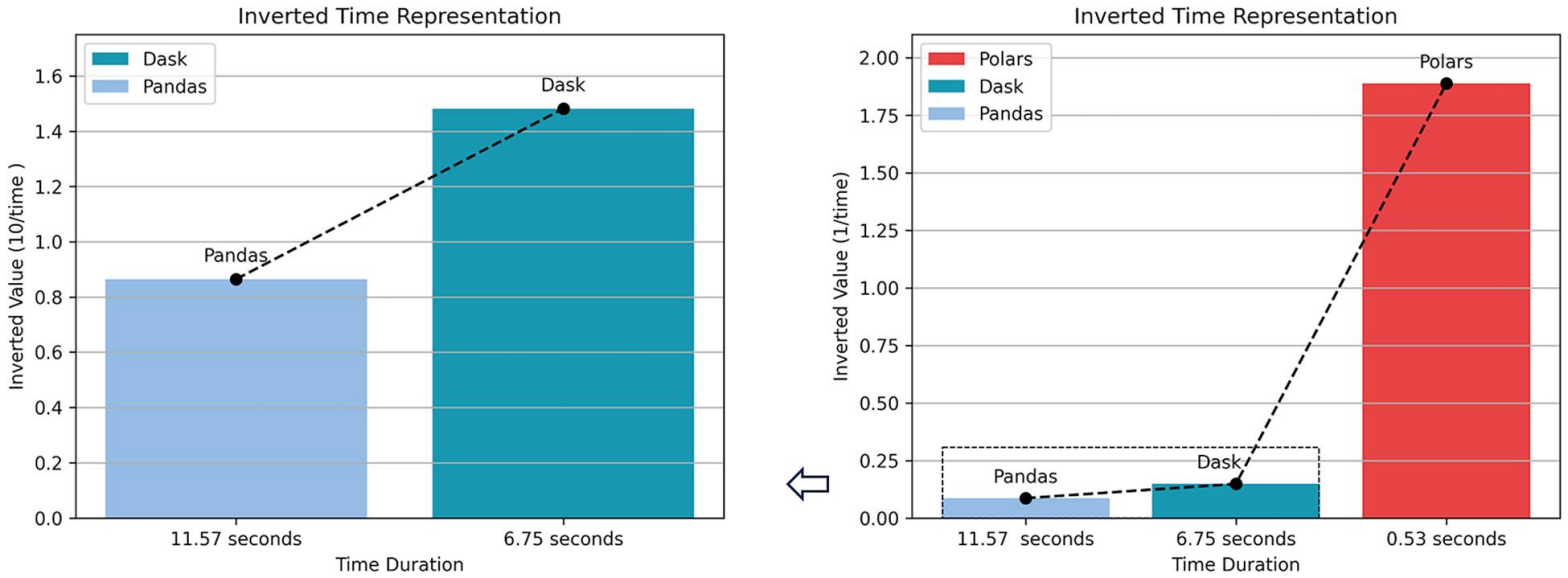
Comparison of experimental results 1.

Results:

As shown in Figure 5, Dask was markedly faster than Pandas, and Polars further reduced execution time, indicating superior performance for large-scale data processing.

#### 5.1.2 Single-file high-frequency retrieval test

We selected P001.csv (10,680,001 records) and repeated the query 100 times to evaluate stability and efficiency under high-frequency use. The same query condition was used: annotation = “7,030 sleeping; MET 0.95”. We compared sequential execution with a multi-process implementation.

**Figure 6:**
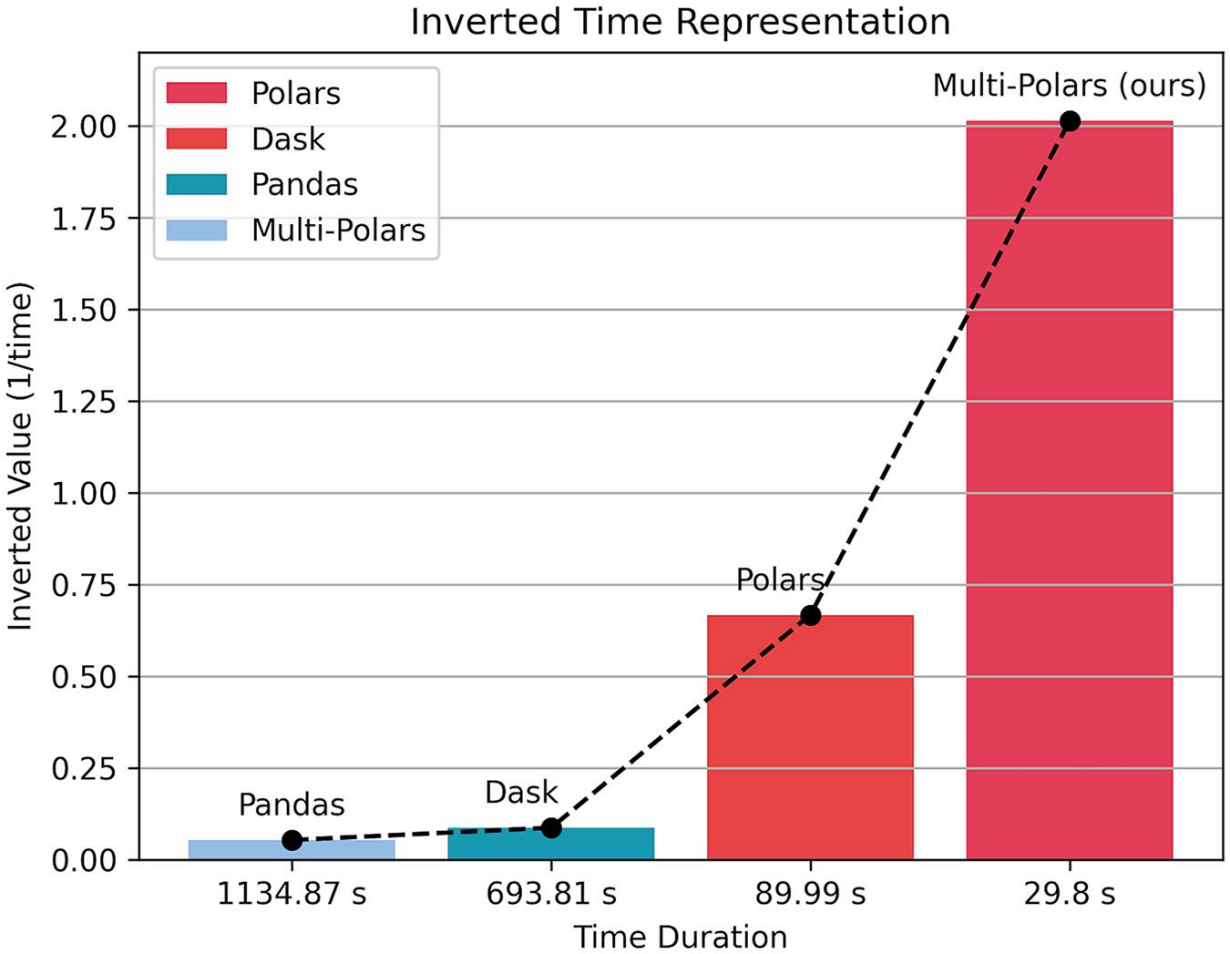
Comparison of experimental results 2.

The result is shown in Figure 6. Compared with the sequential version, multi-process polar has achieved nearly three times the acceleration, significantly reducing the running time.

Summary:(1) For single-file processing, Polars achieved an approximately 22× speedup over traditional approaches;(2) With multi-process parallelism, overall efficiency improved by about 3×;(3) Polars remained advantageous even on relatively low-end hardware. Therefore, all subsequent large-scale data processing and feature engineering were implemented in Polars.

### 5.2 MET prediction model evaluation

To systematically evaluate the effectiveness of the engineered features for MET prediction, we conducted regression experiments under a unified data-processing and modeling framework. First, we built a feature dataset using the statistical and frequency-domain features extracted in Section 4.4.2. To reduce the impact of differences in feature scales on learning, we normalized all features as follows:

$$x_{i}^{\prime }=\frac{x_{i}-\min(x)}{\max(x)-\min(x)},$$ (36)

$x_{i}$ is the i-th data point in the original data, min(x) is the minimum value, max(x) is the maximum value in the dataset, and $x_{i}^{\prime }$ is the normalized i-th data point.

The dataset was split into training and test sets using an 8:2 subject-level split. Specifically, data from 80 participants were used for model training and hyperparameter tuning, while data from the remaining 20 participants were reserved for independent evaluation.This subject-level partition ensures that data from the same individual do not appear in both training and test sets, thereby preventing potential data leakage caused by temporal or window-level correlations.

For model selection, we chose three representative tree-based models: XGBoost, Random Forest, and LightGBM. To avoid bias from manual tuning, we used random search for hyperparameter optimization for all three models. Under the same search space and number of iterations, we obtained approximately optimal parameter settings for each model, ensuring consistent and fair comparisons across algorithms.

Random Forest regression: Given a dataset where denotes input features (statistical and frequency-domain features of acceleration) and denotes the target MET value, the Random Forest prediction can be expressed as:

1. Training multiple decision trees:
  For each tree $T_{k}$, its prediction is ${\hat{y}}_{k}(x)$, where x is a new input sample.
2. Ensemble prediction: The final Random Forest prediction is the average of predictions from all trees:
  $${\hat{y}}_{\mathrm{RF}}(x)=\frac{1}{K}\sum_{k=1}^{K}{\hat{y}}_{k}(x),$$ (37)
  where K is the total number of trees (forest size). ${\hat{y}}_{k}(x)$is the prediction of the k-th tree for the input x.

LightGBM regression is built on the gradient boosted decision tree (GBDT) algorithm, which aims to minimize a loss function through an ensemble of decision trees. Loss optimization: Using gradient descent, GBDT minimizes the loss function:

$$\hat{\;f}(x)=\arg\min\sum_{i=1}^{n}L\;\left(y_{i},f(x_{i})\right)+\Omega (f),$$ (38)

L is the loss function and Ω(f) is a regularization term for model complexity. These models were used to capture the nonlinear mapping between the feature space and MET values, and their performance differences mainly come from different ensembling and optimization strategies.

We evaluated predictive performance using the following metrics:

MAPE (mean absolute percentage error):

$$\text{MAPE}=\frac{1}{n}\sum_{i=1}^{n}\left|\frac{{\hat{y}}_{i}-y_{i}}{y_{i}}\right|\times 100\%.$$ (39)

MSE (mean squared error):

$$\text{MSE}=\frac{1}{n}\sum_{i=1}^{n}({\hat{y}}_{i}-y_{i}{)}^{2},$$ (40)

where $y_{i}$ is the true MET value, ${\hat{y}}_{i}$ is the predicted value, and n is the number of samples.

The test-set results of the different models are shown in Table 10.

**Table 10:** Model comparison results.

Evaluation metrics/models	XGBoost	RandomForest	LightGBM
MAPE	0.2895	0.2914	0.2913
MSE	0.6032	0.6130	0.6128

The results indicate that the three regression models achieved similar overall performance, suggesting that the statistical and frequency-domain features can stably represent MET intensity across activity states. Among the models, XGBoost achieved the best results on both MAPE and MSE, reflecting its advantage in modeling nonlinear features and fitting complex relationships. However, overall differences among models were limited, indicating that the performance ceiling is mainly constrained by data distribution and feature representation rather than the choice of a single regression algorithm. Overall, these results confirm that the proposed feature representation can reliably support MET intensity level estimation from wrist accelerometer signals.

**Figure 7:**
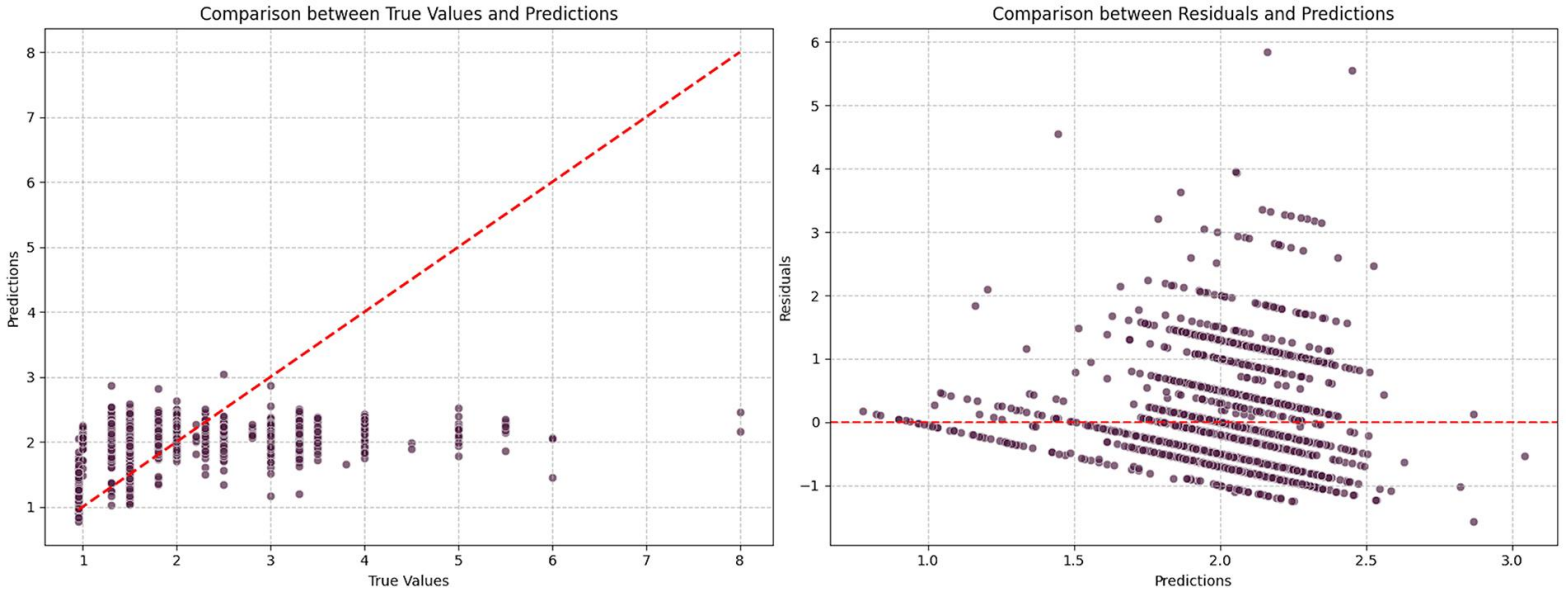
Forecast comparison chart vs. residual plot (XGBoost).

From Figure 7, predictions align well with ground truth in the low-to-moderate MET range. In the high-MET range, prediction errors increase substantially, which is likely due to the small proportion of high-intensity samples, limiting the model’s learning capacity in this range.

### 5.3 Sleep stage identification results using differencing and adaptive thresholds

To validate the method proposed in Section 4.5.1 for sleep-stage identification based on differencing and adaptive thresholds, we visualized the raw acceleration signal and its first-order differenced series, as shown in the figure below:

From the raw time series, the signal shows a stable state with low amplitude and slow variation during sleep; short-term fluctuations appear during body movements or sleep-stage transitions. After first-order differencing, these changes appear as clear peak structures in the differenced series, making behavioral state changes more salient.

**Figure 8:**
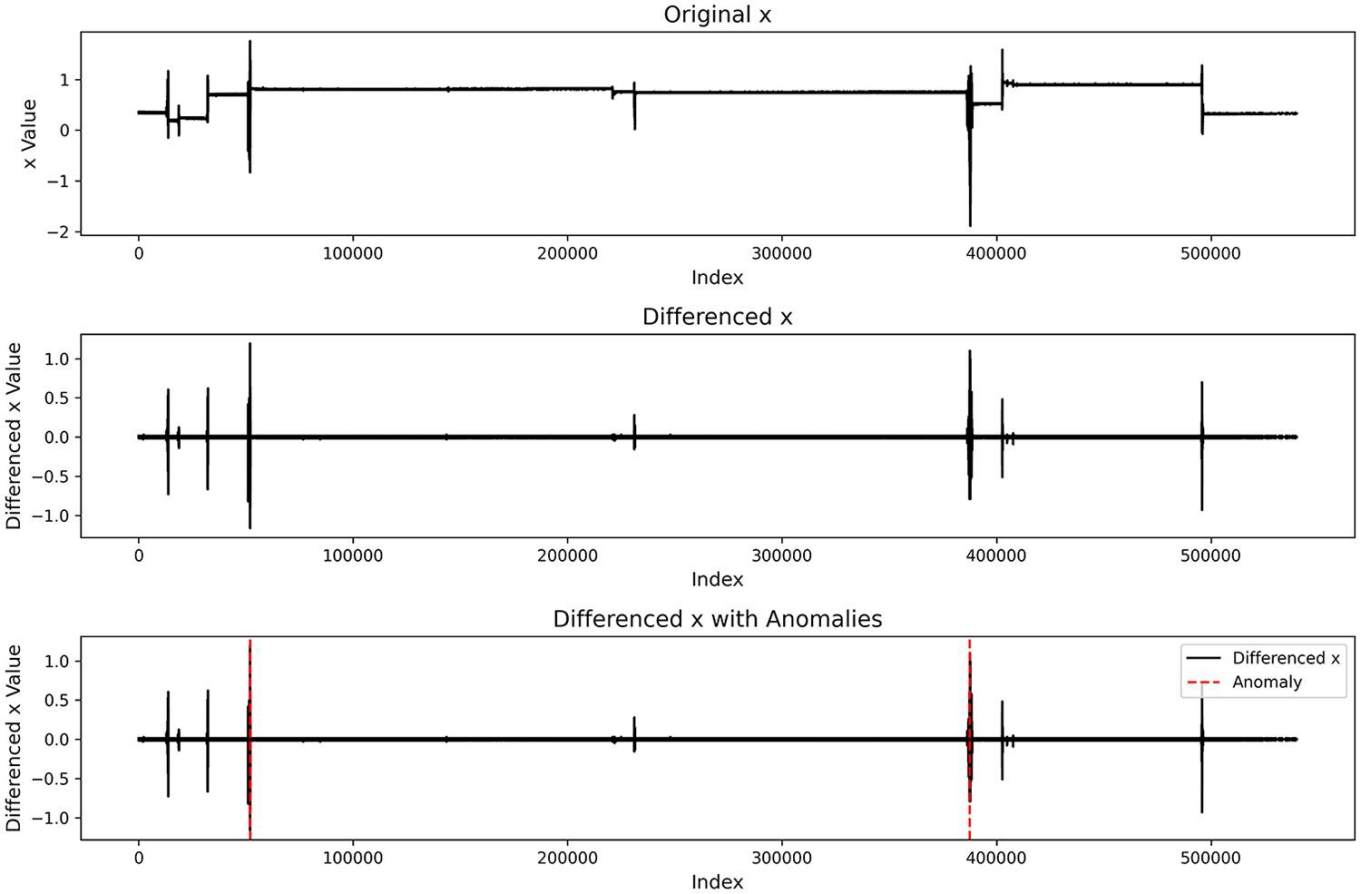
Visualization of adaptive threshold segmentation. The dashed lines indicate detected transition points between coarse sleep states, including quiet sleep segments, transition periods, and movement-related wake-like intervals inferred from accelerometer activity levels.

On this basis, applying an adaptive threshold to segment the differenced signal can automatically distinguish stable intervals from active intervals, enabling coarse-grained sleep-stage partitioning. The anomalous or abrupt-change locations marked by dashed lines in the Figure 8 align well with potential sleep-state transition time points.

To further quantify and summarize the sleep-stage segmentation results in Figure 8, we computed the total sleep duration for 20 volunteers and the cumulative durations of different sleep patterns (Pattern 1 to Pattern 7). The results are shown in Table 11 and can be used to compare inter-individual differences in sleep structure and to verify the stability of the algorithm outputs.

**Table 11:** Sleep stage identification form.

Participant ID	Total sleep (h)	Pattern 1 (h)	Pattern 2 (h)	Pattern 3 (h)	Pattern 4 (h)	Pattern 5 (h)	Pattern 6 (h)	Pattern 7 (h)
P101	4.6667	1.2077	0.4696	0.5305	0.9588	4.6667	1.1329	0.3672
P102	5.8000	0.6956	2.6264	0.9779	0.1444	0.9322	0.4235	–
P103	7.8333	1.6475	3.1047	1.0812	0.0859	1.9141	–	–
P104	6.5833	1.7931	1.7666	1.5238	0.6502	0.8496	–	–
P105	8.1667	1.1311	1.4456	1.2967	0.7933	0.5858	2.9142	–
P106	6.9000	0.6024	0.8895	1.0081	1.5604	2.8396	–	–
P107	9.3333	1.8776	1.0910	0.8116	0.5532	2.7764	2.2235	–
P108	9.3333	1.8776	1.0910	0.8116	0.5532	2.7764	2.2235	–
P109	10.5833	2.0031	2.6303	2.3884	3.5612	–	–	–
P110	6.5000	1.5364	1.4637	1.1821	1.3111	1.0067	–	–
P111	5.1667	0.8732	0.8986	1.8949	1.2766	0.2234	–	–
P112	6.2500	1.8667	2.0499	0.8517	1.4817	–	–	–
P113	3.8333	0.8589	1.2372	0.2472	1.3930	0.1070	–	–
P114	5.3349	3.1678	1.3322	0.2822	0.5527	–	–	–
P115	4.5000	2.0288	0.4712	0.2986	1.1813	0.5201	–	–
P116	4.2667	1.5898	1.1769	0.7470	0.7530	–	–	–
P117	4.5000	0.8932	1.6183	0.3219	0.2892	1.3774	–	–
P118	4.6500	0.4968	2.2794	0.3739	0.7059	0.7940	–	–
P119	4.6667	1.6912	1.4755	4.6667	1.0874	0.4126	–	–
P120	6.0000	1.3321	0.7169	1.0005	1.4506	0.3245	0.6808	0.4946

These results indicate that: (1) Differencing enhances the detectability of subtle changes in the time series. (2) Adaptive thresholds automatically adjust decision criteria according to inter-individual differences in signal amplitude. (3) Their combination enables effective identification of sleep-stage changes without manual labeling.

From a signal-structure perspective, these visualizations verify the feasibility and stability of the proposed sleep-stage identification method on real wearable data.

### 5.4 Limitation

Several limitations of this study should be acknowledged.

First, the dataset contains recordings from a relatively limited number of participants, which may constrain the generalizability of the trained models to broader populations.Second, the accelerometer signals were collected from wrist-worn devices, which may introduce motion artifacts related to upper-limb movements that are not always directly associated with whole-body energy expenditure.Third, the sleep-stage identification method performs coarse-grained segmentation based on motion changes, rather than clinical sleep-stage classification based on polysomnography.

Future research should incorporate larger datasets, multimodal sensors, and clinically validated sleep annotations to further improve model robustness and physiological interpretability.

## 6 Conclusion

This study focuses on physical activity monitoring using wrist-worn wearable devices and develops a complete analytical framework covering raw accelerometer signal processing, MET prediction, and behavioral health analysis and alerting. To address timestamp inconsistency and missing annotations in large-scale high-frequency time series, we proposed an unsupervised anomaly detection and imputation strategy based on duration statistics and DBSCAN clustering, which produced a continuous MET series along the timeline and provided a reliable data basis for subsequent modeling. For MET prediction, we built regression models using multi-dimensional time-domain, frequency-domain, and inter-axis correlation features, and systematically evaluated three tree-based models (XGBoost, Random Forest, and LightGBM) under unified experimental settings. The results show that the engineered features can stably represent metabolic intensity across activity states; XGBoost achieved slightly better accuracy than the other models, but overall differences were limited, suggesting that performance is mainly constrained by data distribution and feature representation capacity. For behavioral health analysis, we further proposed a sleep-stage identification method based on differencing and adaptive thresholds, as well as a sliding-window-based sedentary behavior detection and alert model. Experimental results show that differencing features and the adaptive-threshold mechanism effectively improve sensitivity to sleep-stage transitions, and the sedentary alert model can identify discrete sedentary events at the individual level with clear timestamps, demonstrating feasibility and stability in real wearable-data settings. Overall, the proposed framework does not require complex manual labeling, offers good interpretability and engineering scalability, and can support long-term physical activity monitoring and behavioral health management. Future work will integrate multimodal physiological signals and more refined personalized modeling strategies to improve prediction accuracy for high-intensity activities and explore applications in chronic disease risk assessment and personalized health interventions.

## Data Availability

The data presented in the study are deposited in the Zenodo repository and are available at: https://doi.org/10.5281/zenodo.19408869
